# Supplementary material for: Developing a taxonomy to describe offspring outcomes in studies involving pregnant mammals’ exposure to non-tobacco nicotine: A systematic scoping review
Source: PLoS One. 2023 Feb 3;18(2):e0280805. doi: 10.1371/journal.pone.0280805 (PMC9897539; doi:10.1371/journal.pone.0280805)
Supplement: S2 File — Information for citations included in the systematic scoping review. (PDF) [file pone.0280805.s002.pdf]

Citation title	Author
Effect of maternal nicotine/thiocyanate exposure during gestational period upon pituitary, thyroid and parathyroid function/morphology of 1-month-old rat offspring	Abdelhafez 2014
Increased expression of glial fibrillary acidic protein in cerebellum and hippocampus: differential effects on neonatal brain regional acetylcholinesterase following maternal exposure to combined chlorpyrifos and nicotine	Abdel-Rahman 2003
Maternal exposure to nicotine and chlorpyrifos, alone and in combination, leads to persistently elevated expression of glial fibrillary acidic protein in the cerebellum of the offspring in late puberty	Abdel-Rahman 2004
Maternal exposure of rats to nicotine via infusion during gestation produces neurobehavioral deficits and elevated expression of glial fibrillary acidic protein in the cerebellum and CA1 subfield in the offspring at puberty	Abdel-Rahman 2005
Effects of in utero exposure to alcohol, nicotine, and alcohol plus nicotine, on growth and development in rats	Abel 1979
In utero exposure to nicotine and chlorpyrifos alone, and in combination produces persistent sensorimotor deficits and Purkinje neuron loss in the cerebellum of adult offspring rats	Abou-Donia 2006
Hyperactivity and memory/learning deficits evoked by developmental exposure to nicotine and/or ethanol are mitigated by cAMP and cGMP signaling cascades activation	Abreu-Villaca 2018
Prenatal nicotine exposure modifies behavior of mice through early development	Ajarem 1998
An animal model of effects of nicotine exposure on endometrial receptivity and embryo implantation in pregnancy	Akpak 2017
Prenatal nicotine and CNS development	Al-Hachim 1985
Evaluation of emotional behaviors in young offspring of C57BL/6J mice after gestational and/or perinatal exposure to nicotine in six different time-windows	Alkam 2013
Evaluation of cognitive behaviors in young offspring of C57BL/6J mice after gestational nicotine exposure during different time-windows	Alkam 2013
Prenatal nicotine exposure decreases the release of dopamine in the medial frontal cortex and induces atomoxetine-responsive neurobehavioral deficits in mice	Alkam 2017
Reduced placental size and increased apoptosis are associated with prenatal nicotine exposure in rats	Alzu'Bi 2022
alpha-Lipoic acid attenuates transplacental nicotine-induced germ cell and oxidative DNA damage in adult mice	Anto 2016
Prenatal nicotine exposure affects cardiovascular function and growth of the developing fetus	Aoyagi 2020
Prenatal Nicotine Exposure Impairs the Proliferation of Neuronal Progenitors, Leading to Fewer Glutamatergic Neurons in the Medial Prefrontal Cortex	Aoyama 2016
Nicotine-induced changes in the cerebral circulation in ovine fetuses	Arbeille 1992
Nicotine-induced changes in utero-placental and foetal cerebral circulations in ovine foetuses. [French]	Arbeille 1994
[Changes in the utero-placental and fetal cerebral circulations induced by nicotine in the ovine fetus]	Arbeille 1994
Functional nicotinic acetylcholine receptor expression in stem and progenitor cells of the early embryonic mouse cerebral cortex	Atluri 2001
Prenatal nicotine exposure causes differential impairment of thermogenesis and ventilation in neonatal WT and alpha4 KO mice	Avraam 2011
The effects of chronic nicotine exposure on chemosensitivity of medullary 5-HT neurons	Avraam 2012

Prenatal nicotine exposure increases hyperventilation in alpha4-knock-out mice during mild asphyxia	Avraam 2015
Perinatal Nicotine Reduces Chemosensitivity of Medullary 5-HT Neurons after Maturation in Culture	Avraam 2020
Effect of nicotine sulfate on the hemodynamics and acid base balance of chronically instrumented pregnant sheep	Ayromlooi 1981
Gene-environment interactions affect long-term depression (LTD) through changes in dopamine receptor affinity in Snap25 deficient mice	Baca 2013
Chrna5 genotype determines the long-lasting effects of developmental in vivo nicotine exposure on prefrontal attention circuitry	Bailey 2014
Effect of malnutrition and chronic use of nicotine during pregnancy on lung phospholipid concentration in neonate rats. [Portuguese]	Barbosa 1999
Maternal nicotine exposure leads to decreased cardiac protein disulfide isomerase and impaired mitochondrial function in male rat offspring	Barra 2017
Developmental nicotine exposure adversely effects respiratory patterning in the barbiturate anesthetized neonatal rat	Barreda 2015
Gestational nicotine exposure alone or in combination with ethanol down-modulates offspring immune function	Basta 2000
Perinatal nicotine exposure impairs the maturation of glutamatergic inputs in the auditory brainstem	Baumann 2017
The protective effect of melatonin on nicotine-induced myocardial injury in newborn rats whose mothers received nicotine	Baykan 2008
Studies on nicotine absorption during pregnancy. II. The effects of acute heavy doses on mother and neonates	Becker 1966
Vital effects of chronic nicotine absorption and chronic hypoxic stress during pregnancy and the nursing period	Becker 1971
Prenatal exposure to nicotine affects substance p and preprotachykinin-A mRNA levels in newborn rat	Berner 2008
Nicotine- or epinephrine-induced uteroplacental vasoconstriction and fetal growth in the rat	Birnbaum 1994
Role of phentolamine on the length of pregnancy and foetal development in nicotine-treated pregnant rats	Biswas 1977
Differential responses of hippocampal neurons and astrocytes to nicotine and hypoxia in the fetal guinea pig	Blutstein 2013
Sex differences in heart rate variability during sleep following prenatal nicotine exposure in rat pups	Boychuk 2011
Prenatal nicotine exposure alters postnatal cardiorespiratory integration in young male but not female rats	Boychuk 2011
Prenatal nicotine exposure increased susceptibility to electroconvulsive shock (ECS) in adult rats	Britos 1991
Prenatal nicotine exposure induces cardiac adrenergic subsensitivity in adult rats	Britos 1992
Perinatal nicotine exposure eliminates peak in nicotinic acetylcholine receptor response in adolescent rats	Britton 2007
Fetal and neonatal nicotine exposure and postnatal glucose homeostasis: identifying critical windows of exposure	Bruin 2007
Prenatal Nicotine Exposure Impairs Executive Control Signals in Medial Prefrontal Cortex	Bryden 2016
Developmental nicotine exposure elicits multigenerational disequilibria in proBDNF proteolysis and glucocorticoid signaling in the frontal cortices, striata, and hippocampi of adolescent mice	Buck 2019
Developmental nicotine exposure engenders intergenerational downregulation and aberrant posttranslational modification of cardinal epigenetic factors in the frontal cortices, striata, and hippocampi of adolescent mice	Buck 2020

The Intergenerational Transmission of Developmental Nicotine Exposure-Induced Neurodevelopmental Disorder-Like Phenotypes is Modulated by the Chrna5 D397N Polymorphism in Adolescent Mice	Buck 2021
Developmental Nicotine Exposure Alters Synaptic Input to Hypoglossal Motoneurons and Is Associated with Altered Function of Upper Airway Muscles	BulsWollman 2019
Chronic, Episodic Nicotine Alters Hypoglossal Motor Neuron Function at a Critical Developmental Time Point in Neonatal Rats	BulsWollman 2021
Chronic nicotine in utero selectively suppresses hypoxic sensitivity in neonatal rat adrenal chromaffin cells	Buttigieg 2008
Effects of nicotine administration in developing muscle fibers of rats offspring	Calore 2003
Impaired Lung Mitochondrial Respiration Following Perinatal Nicotine Exposure in Rats	Cannon 2016
Perinatal expression of HSP70 and VEGF in neonatal rat lung vessels exposed to nicotine during gestation	Canoz 2006
Modulation of cell adhesion systems by prenatal nicotine exposure in limbic brain regions of adolescent female rats	Cao 2011
Central myelin gene expression during postnatal development in rats exposed to nicotine gestationally	Cao 2013
Gestational nicotine exposure modifies myelin gene expression in the brains of adolescent rats with sex differences	Cao 2013
The Alteration of Neonatal Raphe Neurons by Prenatal-Perinatal Nicotine. Meaning for Sudden Infant Death Syndrome	Cerpa 2015
Prenatal exposure to nicotine stimulates neurogenesis of orexigenic peptide-expressing neurons in hypothalamus and amygdala	Chang 2013
Maternal intake of nicotine and the airway changes in baby mice	Chen 1982
A Scanning Electron-Microscopy (Sem) and Transmission Electron-Microscopy (Tem) Study of Baby Rabbit Lungs Following Maternal Injection of Nicotine	Chen 1984
Maternal nicotine effects on lung collagen gene expression in newborn rats	Chen 2003
Prenatal nicotine exposure does not cause Purkinje cell loss in the developing rat cerebellar vermis	Chen 2003
Effects of maternal nicotine exposure on lung surfactant system in rats	Chen 2005
Effect of prenatal or perinatal nicotine exposure on neonatal thyroid status and offspring growth in rats	Chen 2005
Effects of nicotine exposure during prenatal or perinatal period on cell numbers in adult rat hippocampus and cerebellum: a stereology study	Chen 2006
Nicotine-induced prenatal overexposure to maternal glucocorticoid and intrauterine growth retardation in rat	Chen 2007
Increased Fetal Thymocytes Apoptosis Contributes to Prenatal Nicotine Exposure-induced Th1/Th2 Imbalance in Male Offspring Mice	Chen 2016
Prenatal nicotine exposure increases osteoarthritis susceptibility in male elderly offspring rats via low-function programming of the TGFbeta signaling pathway	Chen 2019
Sex differences and heritability of adrenal steroidogenesis in offspring rats induced by prenatal nicotine exposure	Chen 2022
Influence of developmental nicotine exposure on glutamatergic neurotransmission in rhythmically active hypoglossal motoneurons	Cholanian 2017
Developmental nicotine exposure alters potassium currents in hypoglossal motoneurons of neonatal rat	Cholanian 2017
Maternal nicotine exposure during gestation and lactation induces cardiac remodeling in rat offspring	Chou 2014
Fetal hemodynamic response to maternal intravenous nicotine administration	Clark 1992

Nicotine administration differentially affects gene expression in the maternal and fetal circadian clock	Clegg 1995
Alterations in cholinergic sensitivity of respiratory neurons induced by pre-natal nicotine: a mechanism for respiratory dysfunction in neonatal mice	Coddou 2009
Perinatal exposure to nicotine causes deficits associated with a loss of nicotinic receptor function	Cohen 2005
Methylphenidate Restores Behavioral and Neuroplasticity Impairments in the Prenatal Nicotine Exposure Mouse Model of ADHD: Evidence for Involvement of AMPA Receptor Subunit Composition and Synaptic Spine Morphology in the Hippocampus	Contreras 2022
Prenatal cocaine and/or nicotine exposure in rats: preliminary findings on long-term cognitive outcome and genital development at birth	Cutler 1996
Intrauterine development in the rat following continuous exposure to nicotine from gestational day 6 through 12	Daeninck 1991
Developmental nicotine exposure results in programming of alveolar simplification and interstitial pulmonary fibrosis in adult male rats	Dasgupta 2012
Antenatal nicotine exposure results in programming of aberrant alveolar development and interstitial pulmonary fibrosis in adult male rats	Dasgupta 2012
Formation of immunodeficiency in newborn mice exposed to nicotine during intrauterine development	Demina 2005
Nicotine-induced retardation of chondrogenesis through down-regulation of IGF-1 signaling pathway to inhibit matrix synthesis of growth plate chondrocytes in fetal rats	Deng 2013
Snail/HDAC1/2 mediate skeletal growth retardation in fetuses caused by prenatal nicotine exposure	Deng 2021
Prenatal nicotine exposure induces cardiac fibrosis in adult male offspring. [Chinese]	Dong 2017
Ventral Tegmental Area Dopamine Neurons Firing Model Reveals Prenatal Nicotine Induced Alterations	Dragomir 2017
Prenatal nicotine-exposure alters fetal autonomic activity and medullary neurotransmitter receptors: implications for sudden infant death syndrome	Duncan 2009
Prenatal nicotine exposure selectively affects nicotinic receptor expression in primary and associative visual cortices of the fetal baboon	Duncan 2015
Effects of nicotine exposure on murine mandibular development	Durham 2019
Direct Effects of Nicotine Exposure on Murine Calvaria and Calvarial Cells	Durham 2019
Prenatal nicotine sex-dependently alters adolescent dopamine system development	Dwyer 2019
Sex-specific alterations in GABA receptor-mediated responses in laterodorsal tegmentum are associated with prenatal exposure to nicotine	Eliasen 2020
Prenatal to early postnatal nicotine exposure impairs central chemoreception and modifies breathing pattern in mouse neonates: a probable link to sudden infant death syndrome	Eugenin 2008
Hypoxia recruits a respiratory-related excitatory pathway to brainstem premotor cardiac vagal neurons in animals exposed to prenatal nicotine	Evans 2005
Effect of gestational nicotine treatment on newborn rat retina: a histopathological and morphometric analysis	Evereklioglu 2003
Effect of nicotine exposure during gestation on neonatal rat crystalline lenses	Evereklioglu 2004
Decreased levels of H3K9ac and H3K27ac in the promotor region of ovarian P450 aromatase mediated low estradiol synthesis in female offspring rats induced by prenatal nicotine exposure as well as in human granulosa cells after nicotine treatment	Fan 2019

Fetal and offspring arrhythmia following exposure to nicotine during pregnancy	Feng 2010
Maternal and fetal metabonomic alterations in prenatal nicotine exposure-induced rat intrauterine growth retardation	Feng 2014
Influence of developmental nicotine exposure on the ventilatory and metabolic response to hyperthermia	Ferng 2015
Prenatal exposure to nicotine attenuates stress-induced hyperthermia in 7- to 8-week-old rats upon exposure to a novel environment	Fewell 2001
Prenatal exposure to nicotine impairs protective responses of rat pups to hypoxia in an age-dependent manner	Fewell 2001
Fetal exposure to nicotine does not alter the core temperature response of 7- to 8-week-old rats to intracerebroventricular administration of PGE(1)	Fewell 2002
Prenatal nicotine alters vigilance states and AchR gene expression in the neonatal rat: implications for SIDS	Frank 2001
Prenatal nicotine exposure changes natural and drug-induced reinforcement in adolescent male rats	Franke 2008
ACTH peptides as organizers of neuronal patterns in development: maturation of the rat neuromuscular junction as seen by scanning electron microscopy	Frischer 1985
Recruitment of GABA(A) receptors in chemoreceptor pulmonary neuroepithelial bodies by prenatal nicotine exposure in monkey lung	Fu 2009
Prenatal nicotine exposure alters respiratory long-term facilitation in neonatal rats	Fuller 2009
Postnatal effects of maternal nicotine exposure on the striatal dopaminergic system in rats	Fung 1989
Effects of prenatal nicotine exposure on rat striatal dopaminergic and nicotinic systems	Fung 1989
Postnatal behavioural effects of maternal nicotine exposure in rats	Fung 1998
Brain-wide genetic mapping identifies the indusium griseum as a prenatal target of pharmacologically unrelated psychostimulants	Fuzik 2019
Prenatal nicotinic exposure prolongs superior laryngeal C-fiber-mediated apnea and bradycardia through enhancing neuronal TRPV1 expression and excitation	Gao 2017
Effects of nicotine on murine incisor development	Gartner 1990
Effects of nicotine on tongue development in the CD-1 mouse	Gartner 1997
Developmental nicotine exposure impairs memory and reduces acetylcholine levels in the hippocampus of mice	Gavini 2021
Failure of nicotine to affect development of offspring when administered to pregnant rats	Geller 1959
Sex-linked differences in avoidance learning in the offspring of rats treated with nicotine during pregnancy	Genedani 1983
Prenatal nicotine exposure enhances the trigeminocardiac reflex via serotonin receptor facilitation in brainstem pathways	Gorini 2013
Effect of nicotine exposure during gestation on neonatal rat ovaries	Gorkem 2016
Maternal nicotine exposure induces congenital heart defects in the offspring of mice	Greco 2022
Chronic maternal nicotine exposure alters neuronal systems in the arcuate nucleus that regulate feeding behavior in the newborn rhesus macaque	Grove 2001
Effect of nicotine exposure during pregnancy and lactation on maternal, fetal, and postnatal rat IGF-II profile	Gruslin 2009
Low doses of nicotine-induced fetal cardiovascular responses, hypoxia, and brain cellular activation in ovine fetuses	Guan 2009
Transient in utero nicotine exposure stimulates mechanosensory-dependent lung development	Gupta 2010

Perinatal nicotine exposure delays genital development in mice	Gyekis 2010
Prenatal nicotine exposure blunts the cardiorespiratory response to hypoxia in lambs	Hafstrom 2002
Altered breathing pattern after prenatal nicotine exposure in the young lamb	Hafstrom 2002
Influence of developmental nicotine exposure on central respiratory-related cholinergic neurotransmission in the brainstem spinal cord preparation	Haggerty 2011
Effect of maternal nicotine on the development of sites for [3H]nicotinebinding in the fetal brain	Hagino 1985
Nicotine reduces embryo growth, delays implantation, and retards parturition in rats	Hammer 1979
The effect of nicotine on endometrial vascular permeability and pregnancy outcome in the rat	Hammer 1981
Effect of nicotine on the metabolic activity of developing rat lung	Hamosh 1978
Effect of nicotine on the development of fetal and suckling rats	Hamosh 1979
Nicotine-induced impairments of spatial cognition and long-term potentiation in adolescent male rats	Han 2014
Gestational IV nicotine produces elevated brain-derived neurotrophic factor in the mesocorticolimbic dopamine system of adolescent rat offspring	Harrod 2011
Offspring of Prenatal IV Nicotine Exposure Exhibit Increased Sensitivity to the Reinforcing Effects of Methamphetamine	Harrod 2012
Gestational exposure to nicotine and/or benzo[a]pyrene causes long-lasting neurobehavioral consequences	Hawkey 2019
Prenatal nicotine exposure induces HPA axis-hypersensitivity in offspringrats via the intrauterine programming of up-regulation of hippocampal GAD67	He 2017
Prenatal nicotine exposure induced high sensitivity of hypothalamic-pituitary-adrenal axis in offspring rats under high fat diet. [Chinese]	He 2017
Cortico-thalamic connectivity is vulnerable to nicotine exposure during early postnatal development through alpha4/beta2/alpha5 nicotinic acetylcholine receptors	Heath 2010
Fetal and neonatal exposure to nicotine in Wistar rats results in increased beta cell apoptosis at birth and postnatal endocrine and metabolic changes associated with type 2 diabetes	Holloway 2005
Rosiglitazone prevents diabetes by increasing beta-cell mass in an animal model of type 2 diabetes characterized by reduced beta-cell mass at birth	Holloway 2008
Effects of maternal nicotine exposure on branching morphogenesis of mouse fetal lung: in vivo and in vitro studies	Hsia 2003
Effects of prenatal nicotine exposure on hepatic glucose and lipid metabolism in offspring rats and its hereditability	Hu 2020
Influence of prenatal nicotine exposure on postnatal development of breathing pattern	Huang 2004
Prenatal nicotine exposure alters the types of nicotinic receptors that facilitate excitatory inputs to cardiac vagal neurons	Huang 2004
Prenatal nicotine exposure recruits an excitatory pathway to brainstem parasympathetic cardioinhibitory neurons during hypoxia/hypercapnia in the rat: implications for sudden infant death syndrome	Huang 2005
Influence of prenatal nicotine exposure on development of the ventilatory response to hypoxia and hypercapnia in neonatal rats	Huang 2010
Maternal nicotine exposure exacerbates neonatal hyperoxia-induced lung fibrosis in rats	Huang 2014
Exposure of nicotine in neonatal rats induces activated inflammatory cytokines and altered biomarkers related to cardiac dysfunction	Huang 2020

Maternal nicotine exposure aggravates metabolic associated fatty liver disease via PI3K/Akt signaling in adult offspring mice	Huang 2021
Nicotine injection during gestation: impairment of reproduction, fetal viability, and development	Hudson 1972
Brain development in offspring of rats treated with nicotine during pregnancy	Hudson 1973
Remodeled salt appetite in rat offspring by perinatal exposure to nicotine	Hui 2009
The effects of prenatal nicotine and THC E-cigarette exposure on motor development in rats	Hussain 2022
Nicotine dose-concentration relationship and pregnancy outcomes in rat: biologic plausibility and implications for future research	Hussein 2007
Long-term effects of gestational nicotine exposure and food-restriction on gene expression in the striatum of adolescent rats	Ilott 2014
Teratogenic effects of nicotine on rat skin	Inaloz 2000
Developmental nicotine exposure enhances inhibitory synaptic transmission in motor neurons and interneurons critical for normal breathing	Jaiswal 2016
Effects of Maternal Nicotine Exposure on Expression of Collagen Type IV and its Roles on Pulmonary Bronchogenesis and Alveolarization in Newborn Mice	Jalali 2010
Fibronectin regulation by vitamin c treatment in kidneys of nicotinic mice offspring	Jalali 2014
Facilitation of cortico-amygdala synapses by nicotine: activity-dependent modulation of glutamatergic transmission	Jiang 2008
Maternal nicotine effects on vascular endothelial growth factor expression and morphometry in rat lungs	Jiang 2012
Nicotine Exposure Causes GATA4 and Tbx5 Gene Repression by DNA Hypermethylation during Cardiac Myogenesis	Jiang 2015
Inhibition of Gata4 and Tbx5 by Nicotine-Mediated DNA Methylation in Myocardial Differentiation	Jiang 2017
Cholinergic modulation of appetite-related synapses in mouse lateral hypothalamic slice	Jo 2005
Developmental nicotine exposure and masculinization of the rat preoptic area	Joglekar 2022
Behavioral effects of prenatal exposure to nicotine in guinea pigs	Johns 1982
The effects of chronic prenatal exposure to nicotine on the behavior of guinea pigs (Cavia porcellus)	Johns 1993
Enhanced susceptibility of CA3 hippocampus to prenatal nicotine exposure	Kalejaiye 2017
Prenatal nicotine exposure alters the nicotinic receptor subtypes that modulate excitation of parasympathetic cardiac neurons in the nucleus ambiguus from primarily alpha3beta2 and/or alpha6betaX to alpha3beta4	Kamendi 2006
Abolishment of serotonergic neurotransmission to cardiac vagal neurons during and after hypoxia and hypercapnia with prenatal nicotine exposure	Kamendi 2009
Gestational nicotine exposure attenuates nicotine-stimulated dopamine release in the nucleus accumbens shell of adolescent Lewis rats	Kane 2004
Nicotine acts on growth plate chondrocytes to delay skeletal growth through the alpha7 neuronal nicotinic acetylcholine receptor	Kawakita 2008
Comparison between dopaminergic and non-dopaminergic neurons in the VTA following chronic nicotine exposure during pregnancy	Keller 2019
Consequences of nicotine exposure during different phases of rat brain development	KhannaSood 2012
[The effect of antenatal nicotine exposure on the male germ cells in Wistar rats]	Khil'kevich 1993

Prenatal nicotine exposure increases frequency and duration of apneic events in neonatal rats	Kidder 2012
Is nicotine depression of neonatal motor behavior exerted through ACTH release?	King 1988
Differential effects of prenatal and postnatal ACTH or nicotine exposure on 5-HT high affinity uptake in the neonatal rat brain	King 1991
Some acute effects of smoking in sheep and their fetuses	Kirschbaum 1970
In Utero Nicotine Exposure Disrupts Global Parathyroid Hormone-Related Protein Signaling	Krebs 2010
Evidence for in vivo nicotine-induced alveolar interstitial fibroblast-to-myofibroblast transdifferentiation	Krebs 2010
Maternal nicotine administration and fetal brain stem damage: a rat model with implications for sudden infant death syndrome	Krous 1981
Prenatal IV nicotine exposure produces a sex difference in sensorimotor gating of the auditory startle reflex in adult rats	Lacy 2011
Intravenous gestational nicotine exposure results in increased motivation for sucrose reward in adult rat offspring	Lacy 2012
IV prenatal nicotine exposure increases the reinforcing efficacy of methamphetamine in adult rat offspring	Lacy 2014
Prenatal nicotine exposure increases heart susceptibility to ischemia/reperfusion injury in adult offspring	Lawrence 2008
In Utero Exposure to Nicotine Reduces PKC epsilon Gene Expression in the Fetal Rat Heart	Lawrence 2010
Foetal nicotine exposure causes PKCepsilon gene repression by promoter methylation in rat hearts	Lawrence 2011
Effects of nicotine on pulmonary surfactant proteins A and D in ovine lung epithelia	Lazic 2010
Effects of caffeine and nicotine administration on growth and ossification of the ICR mouse fetus	Leblebicioglu-Bekcioglu 1995
Effects of maternal intravenous nicotine administration on locomotor behavior in pre-weanling rats	LeSage 2006
Prenatal nicotine exposure and cognitive performance in rats	Levin 1993
Prenatal nicotine effects on memory in rats: pharmacological and behavioral challenges	Levin 1996
Increased nicotine self-administration following prenatal exposure in female rats	Levin 2006
Perinatal nicotine exposure increases vulnerability of hypoxic-ischemic brain injury in neonatal rats: role of angiotensin II receptors	Li 2012
Exposure to nicotine during pregnancy and altered learning and memory in the rat offspring	Li 2015
Effect of nicotine exposure during maternal pregnancy on organ development of offspring mice. [Chinese]	Li 2015
Prenatal nicotine affects fetal testosterone and sexual dimorphism of saccharin preference	Lichtensteiger 1985
Changes in the glutamate release and uptake of cerebellar cells in perinatally nicotine-exposed rat pups	Lim 2001
Chronic exposure of nicotine modulates the expressions of the cerebellar glial glutamate transporters in rats	Lim 2003
Prenatal nicotine exposure induced a hypothalamic-pituitary-adrenal axis-associated neuroendocrine metabolic programmed alteration in intrauterine growth retardation offspring rats	Liu 2012
Nicotine Suppressed Fetal Adrenal StAR Expression via YY1 Mediated-Histone Deacetylation Modification Mechanism	Liu 2016
alpha7 nAChR mediated Fas demethylation contributes to prenatal nicotineexposure-induced programmed thymocyte apoptosis in mice	Liu 2017

Melatonin ameliorates murine fetal oocyte meiotic dysfunction in F1 and F2 offspring caused by nicotine exposure during pregnancy	Liu 2020
Deletion of alpha2* nicotinic acetylcholine receptors ablates learning and memory in maternal nicotine treated adolescent offspring	Lotfipour 2017
Effects of Nicotine Administration Throughout Pregnancy on Fetal Size, Placental Weight and Fetal and Maternal Endocrine Parameters at Term	Louis 1981
Prenatal nicotine exposure increases the strength of GABA(A) receptor-mediated inhibition of respiratory rhythm in neonatal rats	Luo 2004
Prenatal nicotine exposure alters glycinergic and GABAergic control of respiratory frequency in the neonatal rat brainstem-spinal cord preparation	Luo 2007
The effect of prenatal nicotine on expression of nicotine receptor subunits in the fetal brain	Lv 2008
Influence of exposure to nicotine during pregnancy on the learning and memory for adult offspring	Ma 2018
Effects of maternal nicotine exposure on expression of laminin alpha 5 in lung tissue of newborn	MahdiShariati 2012
Prenatal nicotine alters maturation of breathing and neural circuits regulating respiratory control	Mahliere 2008
Galantamine improves enhanced impulsivity, impairments of attention and long-term potentiation induced by prenatal nicotine exposure to mice	Mamiya 2020
The effect of nicotine on fetal breathing movements in conscious pregnantewes	Manning 1978
Fetal nicotine exposure increases preference for nicotine odor in early postnatal and adolescent, but not adult, rats	Mantella 2013
The effect of prenatal nicotine on mRNA of central cholinergic markers and hematological parameters in rat fetuses	Mao 2008
Prenatal exposure to nicotine with associated in utero hypoxia decreased fetal brain muscarinic mRNA in the rat	Mao 2008
Effect of maternal nicotine exposure on neonatal lung elastic tissue and possible consequences	Maritz 1992
The influence of maternal nicotine exposure on the interalveolar septal status of neonatal rat lung	Maritz 1994
Biomedical response of neonatal rat lung to maternal nicotine exposure	Maritz 1995
The influence of maternal nicotine exposure on the status of the connective tissue framework of developing rat lung	Maritz 1996
Growth, development and activity in rat offspring following maternal drug exposure	Martin 1976
Combined exposure to nicotine and ethanol throughout full gestation results in enhanced acquisition of nicotine self-administration in young adult rat offspring	Matta 2007
Prenatal nicotine is associated with reduced AMPA and NMDA receptor-mediated rises in calcium within the laterodorsal tegmentum: a pontine nucleus involved in addiction processes	McNair 2015
Prenatal nicotine exposure evokes changes in the incidence and degree of fetal electrocortical activation	McNerney 1993
The effects of perinatal exposure to nicotine on plasma LH levels in prepubertal rats	Meyer 1987
Maternal Nicotine Induces Collagen Type IV Changes in the Mice Lung Parenchyma and its Vessels	Mohammadi 2011
Prenatal exposure to nicotine in pregnant rat increased inflammatory marker in newborn rat	Mohsenzadeh 2014
Intravenous prenatal nicotine exposure increases orexin expression in the lateral hypothalamus and orexin innervation of the ventral tegmental area in adult male rats	Morgan 2013

Resistance of rat fetuses to nicotine-induced lipolysis	Mosier 1974
Prenatal nicotine exposure alters neuroanatomical organization of the developing brain	Muhammad 2012
Training on motor and visual spatial learning tasks in early adulthood produces large changes in dendritic organization of prefrontal cortex and nucleus accumbens in rats given nicotine prenatally	Muhammad 2013
Prenatal nicotine exposure affects the development of the central serotonergic system as well as the dopaminergic system in rat offspring: involvement of route of drug administrations	Muneoka 1997
Prenatal administration of nicotine results in dopaminergic alterations in the neocortex	Muneoka 1999
Nicotine exposure during pregnancy is a factor which influences serotonin transporter density in the rat brain	Muneoka 2001
Effects of ethanol and nicotine co-administration on follicular atresia and placental histo-morphology in the first-generation mice pups during intrauterine development and lactation periods	Musanejad 2021
Does prenatal nicotine exposure alter the brain's response to nicotine in adolescence? A neuroanatomical analysis	Mychasiuk 2013
Long-term alterations to dendritic morphology and spine density associated with prenatal exposure to nicotine	Mychasiuk 2013
Environmental enrichment alters structural plasticity of the adolescent brain but does not remediate the effects of prenatal nicotine exposure	Mychasiuk 2014
[The effects of nicotine on the synthesis and secretion of surfactant in the rabbit fetal lung]	Nakamura 1988
Neuroprotective effect of vitamin C against the ethanol andnicotinemodulation of GABA(B) receptor and PKA-alpha expression in prenatal rat brain	Naseer 2010
Influence of nicotine and caffeine on skeletal development in the rat	Nash 1989
Perinatal effects of nicotine	Nasrat 1986
Prenatal exposure to nicotine via maternal infusions: effects on development of catecholamine systems	Navarro 1988
Effects of prenatal nicotine exposure on development of central and peripheral cholinergic neurotransmitter systems. Evidence for cholinergic trophic influences in developing brain	Navarro 1989
Prenatal exposure to nicotine impairs nervous system development at a dose which does not affect viability or growth	Navarro 1989
Prenatal nicotine exposure impairs beta-adrenergic function: persistent chronotropic subsensitivity despite recovery from deficits in receptor binding	Navarro 1990
Effects of fetal nicotine exposure on development of adrenergic receptor binding in rat brain regions: Selective changes in alpha1-receptors	Navarro 1990
Prenatal Nicotine Exposure Induces Low Birthweight and Hyperinsulinemia in Male Rats	Nemoto 2021
Maternal nicotine induces collagen type IV changes and its role on pulmonary bronchogenesis and alveolarization in mouse newborns. [Arabic]	Nikravesh 2010
Developmental anomalies in offspring of pregnant mice treated with nicotine	Nishimura 1958
Prenatal exposure to nicotine in mice is associated with alterations in development and cellular and synaptic effects of alcohol in a brainstem arousal nucleus	Nunes-Freitas 2021
Prenatal cholinergic stimulation of pulmonary neuroendocrine cells by nicotine	Nylen 1988
Modulation of pulmonary bombesin by nicotine and vagotomy	Nylen 1990
Prenatal Nicotine Exposure Augments Renal Oxidative Stress in Embryos of Pregnant Rats with Reduced Uterine Perfusion Pressure	Ojeda 2016

Impact of prenatal nicotine on the structure of midbrain dopamine regions in the rat	Omelchenko 2016
Morphological and neurohistological changes in adolescent rats administered with nicotine during intrauterine life	Omotoso 2013
Neuromorphometric analysis of the lateralprefrontal cortex of young wistar rats exposed to nicotine in utero	Omotoso 2014
Histoenzymic evaluation of the frontal cortex of young Wistar rats following prenatal nicotine administration	Omotoso 2015
Prenatal Exposure to Gestational Nicotine before Neurulation is Detrimental to Neurodevelopment of Wistar Rats' Offspring	Omotoso 2018
Alterations of brain tissue in fetal rats exposed to nicotine in utero: possible involvement of nitric oxide and catecholamines	Onal 2004
Influence of prenatal administration of nicotine/thiocyanate on the morphology of exocrine pancreas of 1-month-old rat offspring	Othman 2020
Testosterone and testicular changes in F1 offspring of Wistar ratmaternally exposed to nicotine during gestation	Oyeyipo 2018
Does maternal nicotine exposure during gestation increase the injury severity of small intestine in the newborn rats subjected to experimental necrotizing enterocolitis	Ozkan 2005
Nicotine Exposure During Pregnancy Results in Persistent Midline Epithelial Seam With Improper Palatal Fusion	Ozturk 2016
Fibronectin regulation by Vitamin C treatment in kidneys of nicotinic mice offspring	Pahang 2014
Developmental nicotine exposure induced alterations in behavior and glutamate receptor function in hippocampus	Parameshwaran 2012
Long term alterations in synaptic physiology, expression of beta2 nicotinic receptors and ERK1/2 signaling in the hippocampus of rats with prenatal nicotine exposure	Parameshwaran 2013
Gestational nicotine-induced changes in adolescent neuronal activity	Park 2006
Alcohol and smokeless tobacco effects on the CD-1 mouse fetus	Paulson 1992
In utero nicotine exposure causes persistent, gender-dependant changes in locomotor activity and sensitivity to nicotine in C57Bl/6 mice	Pauly 2004
Prenatal exposure to nicotine modifies kidney weight and blood pressure in genetically susceptible rats: a case of gene-environment interaction	Pausova 2003
Vitamin B12 reduces the negative effects of nicotine on fetal bone development in the rats	Payas 2022
Behavioral teratogenicity induced by nonforced maternal nicotineconsumption	Paz 2007
Excitotoxicity and compensatory upregulation of GAD67 in fetal rat hippocampus caused by prenatal nicotine exposure are associated with inhibition of the BDNF pathway	Pei 2019
Postnatal effects of maternal nicotine exposure	Peters 1979
Sex-dependent biological changes following prenatal nicotine exposure in the rat	Peters 1982
The effects of totigestational exposure to nicotine on pre- and postnatal development in the rat	Peters 1982
Prenatal nicotine exposure increases adrenergic receptor binding in the rat cerebral cortex	Peters 1984
Chronic nicotinic exposure decreases the synaptic strength of nAChR-mediated glutamatergic input onto neonatal hypoglossal motoneurons	Pilarski 2010
Atomoxetine Reestablishes Long Term Potentiation in a Mouse Model of Attention Deficit/Hyperactivity Disorder	Pina 2020
Effects of ethanol, nicotine and caffeine gestational exposure of female rats on lung and brain tissues in fetuses: morphological and biological study	Pintican 2019

Exposure to threshold doses of nicotine in utero: I. Neuroendocrine response to restraint stress in adult male offspring	Poland 1994
Exposure to threshold doses of nicotine in utero: II. Neuroendocrine response to nicotine in adult male offspring	Poland 1994
Exposure to threshold doses of nicotine in utero: III. Augmentation of the prolactin and ACTH response to 8-OH DPAT by desipramine treatment is compromised in adult male offspring	Poland 1996
Prenatal nicotine exposure alters postsynaptic AMPA receptors and glutamate neurotransmission within the laterodorsal tegmentum (LDT) of juvenile mice	Polli 2018
Alterations in NMDAR-mediated signaling within the laterodorsal tegmental nucleus are associated with prenatal nicotine exposure	Polli 2019
Prenatal nicotine exposure in mice induces sex-dependent anxiety-like behavior, cognitive deficits, hyperactivity, and changes in the expression of glutamate receptor associated-genes in the prefrontal cortex	Polli 2020
Cellular and Molecular Changes in Hippocampal Glutamate Signaling and Alterations in Learning, Attention, and Impulsivity Following Prenatal Nicotine Exposure	Polli 2020
Prenatal exposure to nicotine: effects on prepulse inhibition and central nicotinic receptors	Popke 1997
Influence of developmental nicotine exposure on spike-timing precision and reliability in hypoglossal motoneurons	Powell 2015
Developmental nicotine exposure disrupts dendritic arborization patterns of hypoglossal motoneurons in the neonatal rat	Powell 2016
Vitamin C prevents the effects of prenatal nicotine on pulmonary function in newborn monkeys	Proskocil 2005
Prenatal nicotine exposure induces thymic hypoplasia in mice offspring from neonatal to adulthood	Qu 2019
Prenatal nicotine exposure leads to decreased histone H3 lysine 9 (H3K9) methylation and increased p66shc expression in the neonatal pancreas	Raez-Villanueva 2021
Prenatal nicotine exposure leads to decreased histone H3 lysine 9 (H3K9) methylation and increased p66shc expression in the neonatal pancreas	Raez-Villanueva 2022
Optical coherence tomography angiography to evaluate murine fetal brain vasculature changes caused by prenatal exposure to nicotine	Raghunathan 2020
Erratum: In utero nicotine exposure alters fetal rat lung alveolar type II cell proliferation, differentiation, and metabolism (American Journal of Physiology - Lung Cellular and Molecular Physiology (2007) 292, (L323-L333) DOI: 10.1152/ajplung.00071.2006)	Rehan 2007
Effects of acute and chronic prenatal nicotine treatment on central catecholamine systems of male and female rat fetuses and offspring	Ribary 1989
Hyperactivity in the offspring of nicotine-treated rats: role of the mesolimbic and nigrostriatal dopaminergic pathways	Richardson 1994
The effect of nicotine and alcohol on the fertility and life span of rats. A cytological analysis	Riesenfeld 1987
Prenatal nicotine exposure increases apnoea and reduces nicotinicpotentiation of hypoglossal inspiratory output in mice	Robinson 2002
Prenatal ablation of nicotinic receptor alpha7 cell lineages produces lumbosacral spina bifida the severity of which is modified by choline and nicotine exposure	Rogers 2012
Elevated blood pressure in offspring of rats exposed to diverse chemicals during pregnancy	Rogers 2014
Gender-related response in open-field activity following developmental nicotine exposure in rats	Romero 2004

Response of the Developing Neuromuscular System of the Rat to Nicotineand the Neurotropic Peptide Fragment Acth/Msh 4-10	Rose 1987
Does nicotine alter the metabolic and histochemical profile of developing rat EDL muscle?	Rose 1988
Accelerated neuromuscular development in the rat with prenatal exposure to nicotine	Rose 1990
A sensitive period in gestation for nicotine acceleration of neuromuscular maturation	Rose 1991
Prenatal nicotine exposure and behavior	Rossi 2003
The effect of chronic oral nicotine administration on fetal weight and placental amino acid accumulation in mice	Rowell 1982
Effects of prenatal nicotine exposure on the morphogenesis of somatosensory cortex	Roy 1994
Effects of gestational nicotine exposure on hippocampal morphology	Roy 1998
Prenatal nicotine exposure evokes alterations of cell structure in hippocampus and somatosensory cortex	Roy 2002
Postnatal effects of nicotine on incisor development of albino mouse	Saad 1990
Teratogenic effects of nicotine on palate formation in mice	Saad 1990
Postnatal effects of nicotine on first molar development in the CD-1 mouse	Saad 1991
Teratogenic effects of nicotine on first molar odontogenesis in the mouse	Saad 1991
Altered lung development after prenatal nicotine exposure in young lambs	Sandberg 2004
Prenatal nicotine exposure transiently alters the lung mechanical response to hypoxia in young lambs	Sandberg 2007
Fetal nicotine exposure increases airway responsiveness and alters airway wall composition in young lambs	Sandberg 2011
Postnatal effects of prenatal nicotine exposure on body weight, brain size and cortical connectivity in mice	Santiago 2012
Prenatal nicotine exposure increases anxiety and modifies sensorimotor integration behaviors in adult female mice	Santiago 2014
Adrenal-mediated rather than direct effects of nicotine as a basis of altered sex steroid synthesis in fetal and neonatal rat	Sarasin 2003
A new device for monitoring early motor development: prenatal nicotine-induced changes	Schlumpf 1988
Gestational exposure to nicotine in drinking water: teratogenic effects and methodological issues	Schneider 2010
Prenatal exposure to nicotine impairs performance of the 5-choice serial reaction time task in adult rats	Schneider 2011
Inattentiveness and Impulsivity in Adult Rats Prenatally Exposed to Nicotine	Schneider 2011
Hyperactivity, increased nicotine consumption and impaired performance in the five-choice serial reaction time task in adolescent rats prenatally exposed to nicotine	Schneider 2012
Perinatal administration of nicotine alters subsequent sexual behavior and testosterone levels of male rats	Segarra 1989
Fetal nicotine exposure ablates the ability of postnatal nicotine challenge to release norepinephrine from rat brain regions	Seidler 1992
Prenatal nicotine increases pulmonary alpha7 nicotinic receptor expression and alters fetal lung development in monkeys	Sekhon 1999
Prenatal nicotine exposure alters pulmonary function in newborn rhesus monkeys	Sekhon 2001
Maternal nicotine exposure upregulates collagen gene expression in fetal monkey lung. Association with alpha7 nicotinic acetylcholine receptors	Sekhon 2002

Prenatal nicotine exposure increases connective tissue expression in foetal monkey pulmonary vessels	Sekhon 2004
Expression of lynx1 in developing lung and its modulation by prenatal nicotine exposure	Sekhon 2005
Exposure to nicotine during gestation interferes with the colonization of fetal bone marrow by hematopoietic stem/progenitor cells	Serobyan 2005
Effects of prenatal administration of nicotine on amino acid pools, protein metabolism, and nicotine binding in the brain	Sershen 1982
Prenatal nicotine sex-dependently alters agonist-induced locomotion and stereotypy	Shacka 1997
Exposure to prenatal nicotine transiently increases neuronal nicotinicreceptor subunit alpha7, alpha4 and beta2 messenger RNAs in the postnatal rat brain	Shacka 1998
Different ideas associated renal malformation and laminin alpha5 expression caused by maternal nicotine exposures	ShariatiKohbanani 2016
Postnatal development of rat progeny after antenatal nicotine exposure	Sheveleva 1984
Epigenomic and metabolic responses of hypothalamic POMC neurons to gestational nicotine exposure in adult offspring	Silva 2016
Effect of prenatal nicotine exposure on biphasic hypoxic ventilatory response and protein kinase C expression in caudal brain stem of developing rats	Simakajornboon 2004
The effect of prenatal nicotine exposure on PDGFR-mediated anti-apoptotic mechanism in the caudal brainstem of developing rat	Simakajornboon 2010
The effect of prenatal nicotine exposure on early apoptotic markers during hypoxia in the caudal brainstem of developing mice	Simakajornboon 2010
Modulation Of Biphasic Hypoxic Ventilatory Response By Specific Serotonin Receptor Antagonist	Simakajornboon 2011
Ascorbic Acid Ameliorates Nicotine Exposure Induced Impaired Spatial Memory Performance in Rats	Sirasanagandla 2014
Effects of maternal nicotine injections on brain development in the rat: ornithine decarboxylase activity, nucleic acids and proteins in discrete brain regions	Slotkin 1986
Development of [3H]nicotine binding sites in brain regions of rats exposed to nicotine prenatally via maternal injections or infusions	Slotkin 1987
Effects of prenatal nicotine exposure on biochemical development of rat brain regions: maternal drug infusions via osmotic minipumps	Slotkin 1987
Fetal nicotine exposure produces postnatal up-regulation of adenylate cyclase activity in peripheral tissues	Slotkin 1990
Chronic prenatal nicotine exposure sensitizes rat brain to acute postnatal nicotine challenge as assessed with ornithine decarboxylase	Slotkin 1991
Altered development of basal and forskolin-stimulated adenylate cyclase activity in brain regions of rats exposed to nicotine prenatally	Slotkin 1992
Impact of fetal nicotine exposure on development of rat brain regions: critical sensitive periods or effects of withdrawal?	Slotkin 1993
Loss of neonatal hypoxia tolerance after prenatal nicotine exposure: implications for sudden infant death syndrome	Slotkin 1995
Cryptic brain cell injury caused by fetal nicotine exposure is associated with persistent elevations of c-fos protooncogene expression	Slotkin 1997
Impaired cardiac function during postnatal hypoxia in rats exposed to nicotineprenatally: implications for perinatal morbidity and mortality, and for sudden infant death syndrome	Slotkin 1997
Cholinergic receptors in heart and brainstem of rats exposed to nicotineduring development: implications for hypoxia tolerance and perinatal mortality	Slotkin 1999

Effects of prenatal nicotine exposure on primate brain development and attempted amelioration with supplemental choline or vitamin C: neurotransmitter receptors, cell signaling and cell development biomarkers in fetal brain regions of rhesus monkeys	Slotkin 2005
Permanent, sex-selective effects of prenatal or adolescent nicotine exposure, separately or sequentially, in rat brain regions: indices of cholinergic and serotonergic synaptic function, cell signaling, and neural cell number and size at 6 months of age	Slotkin 2007
Separate or sequential exposure to nicotine prenatally and in adulthood: persistent effects on acetylcholine systems in rat brain regions	Slotkin 2007
Lasting effects of nicotine treatment and withdrawal on serotonergic systems and cell signaling in rat brain regions: separate or sequential exposure during fetal development and adulthood	Slotkin 2007
Additive and synergistic effects of fetal nicotine and dexamethasone exposure on cholinergic synaptic function in adolescence and adulthood: Implications for the adverse consequences of maternal smoking and pharmacotherapy of preterm delivery	Slotkin 2010
Mimicking maternal smoking and pharmacotherapy of preterm labor: fetal nicotine exposure enhances the effect of late gestational dexamethasone treatment on noradrenergic circuits	Slotkin 2011
Prenatal nicotine exposure in rhesus monkeys compromises development of brainstem and cardiac monoamine pathways involved in perinatal adaptation and sudden infant death syndrome: amelioration by vitamin C	Slotkin 2011
Prenatal nicotine alters the developmental neurotoxicity of postnatal chlorpyrifos directed toward cholinergic systems: better, worse, or just "different?"	Slotkin 2015
Prenatal nicotine changes the response to postnatal chlorpyrifos: Interactions targeting serotonergic synaptic function and cognition	Slotkin 2015
Prenatal drug exposures sensitize noradrenergic circuits to subsequent disruption by chlorpyrifos	Slotkin 2015
Prenatal nicotine increases testosterone levels in the fetus and female offspring	Smith 2003
Interactive effects of prenatal cocaine nd nicotine exposure on maternal toxicity, postnatal development and behavior in the rat	Sobrian 1995
Prenatal cocaine and/or nicotine exposure produces depression and anxiety in aging rats	Sobrian 2003
Prenatal nicotine and/or cocaine differentially alters nicotine-induced sensitization in aging offspring	Sobrian 2008
Prenatal nicotine exposure alters early pancreatic islet and adipose tissue development with consequences on the control of body weight and glucose metabolism later in life	Somm 2008
The effects of prenatal nicotine on radial-arm maze performance in rats	Sorenson 1991
Maternal nicotine depresses eupneic ventilation of neonatal rats	St-John 1999
Prenatal nicotine exposure induced GDNF/c-Ret pathway repression-related fetal renal dysplasia and adult glomerulosclerosis in male offspring	Sun 2015
In utero nicotine exposure epigenetically alters fetal chromatin structure and differentially regulates transcription of the glucocorticoid receptor in a rat model	Suter 2015
Pharmacologic effects of nicotine upon the fetus and mother in the rhesus monkey	Suzuki 1971
Effect of nicotine upon uterine blood flow in the pregnant rhesus monkey	Suzuki 1980
The effect of maternal nicotine on basement membrane collagen IV of brain microvessels changes in neonatal Balb/C mice	Tahajjodi 2014

Angiotensin II-mediated vascular changes in aged offspring rats exposed to perinatal nicotine	Tao 2013
Pup survival and prolactin levels in nicotine-treated lactating rats	Terkel 1973
Prenatal nicotine increases matrix metalloproteinase 2 (MMP-2) expression in fetal guinea pig hearts	Thompson 2011
Prenatal nicotine exposure induces poor articular cartilage quality in female adult offspring fed a high-fat diet and the intrauterine programming mechanisms	Tie 2016
Intrauterine low-functional programming of IGF1 by prenatal nicotineexposure mediates the susceptibility to osteoarthritis in female adult rat offspring	Tie 2016
Hyperactivity induced by prenatal nicotine exposure is associated with an increase in cortical nicotinic receptors	Tizabi 1997
Prenatal nicotine exposure is associated with an increase in [125I]epibatidine binding in discrete cortical regions in rats	Tizabi 2000
Prenatal nicotine exposure: effects on locomotor activity and central [125I]alpha-BT binding in rats	Tizabi 2000
Effect of prenatal exposure to nicotine on kidney glomerular mass and AT1R expression in genetically diverse strains of rats	Toledo-Rodriguez 2012
Does concurrent or prior nicotine exposure interact with neonatal hypoxia to produce cardiac cell damage?	Tolson 1995
In-utero exposure to nicotine alters the development of the rabbit cardiac conduction system and provides a potential mechanism for sudden infantdeath syndrome	Ton 2017
Persistent c-fos induction by nicotine in developing rat brain regions: interaction with hypoxia	Trauth 1999
Perinatal nicotine exposure alters lung development and induces HMGB1-RAGE expression in neonatal mice	Tsai 2021
An assessment of the long-term developmental and behavioral teratogenicity of prenatal nicotine exposure	Vaglenova 2004
Long-lasting teratogenic effects of nicotine on cognition: gender specificity and role of AMPA receptor function	Vaglenova 2008
Prenatal nicotine alters nicotinic receptor development in the mouse brain	vandeKamp 1994
Impact of gestational nicotine exposure on intrauterine and fetal infection in a rodent model	vonChamier 2017
Nicotine Induces Maternal and Fetal Inflammatory Responses Which Predispose Intrauterine Infection Risk in a Rat Model	vonChamier 2021
Prenatal nicotine exposure selectively affects perinatal forebrain aromatase activity and fetal adrenal function in male rats	vonZiegler 1991
Prenatal nicotine exposure alters intrinsic properties of neonatal hypoglossal motor neurons in the rhythmic medullary slice preparation	Wakefield 2010
The Effect of Maternal Oral Intake of Nicotine on the Growth and Maturation of Fetal and Baby Mouse Lungs	Wang 1983
The cumulative scanning electron microscopic changes in baby mouse lungs following prenatal and postnatal exposures to nicotine	Wang 1984
Growth retardation of fetal rats exposed to nicotine in utero: possible involvement of CYP1A1, CYP2E1, and P-glycoprotein	Wang 2009
Gestational nicotine exposure regulates expression of AMPA and NMDA receptors and their signaling apparatus in developing and adult rat hippocampus	Wang 2011
Prenatal nicotine and maternal deprivation stress de-regulate the development of CA1, CA3, and dentate gyrus neurons in hippocampus of infant rats	Wang 2013
Prenatal nicotine exposure induces gender-associated left ventricular-arterial uncoupling in adult offspring	Wang 2015
Inhibition of miRNA-210 reverses nicotine-induced brain hypoxic-ischemic injury in neonatal rats	Wang 2017

Protective effects of melatonin against nicotine-induced disorder of mouse early folliculogenesis	Wang 2018
Gestational nicotine treatment modulates cell death/survival-related pathways in the brains of adolescent female rats	Wei 2011
Attenuated cholesterol metabolism pathway suppresses regulatory T cell development in prenatal nicotine exposed female mice	Wen 2019
Asthma susceptibility in prenatal nicotine-exposed mice attributed to beta-catenin increase during CD4⁺ T cell development	Wen 2022
Perinatal nicotine treatment induces transient increases in NACHO protein levels in the rat frontal cortex	Wichern 2017
Perinatal nicotine attenuates the hypoxia-induced up-regulation of tyrosine hydroxylase and galanin mRNA in locus ceruleus of the newborn mouse	Wickstrom 2002
Maternal adipose tissue response to nicotine administration in the pregnant rat: effects on fetal body fat and cellularity	Williams 1984
Effects of exposure to nicotine and to sidestream smoke on pregnancy outcome in rats	Witschi 1994
Developmental plasticity of GABAergic neurotransmission to brainstem motoneurons	Wollman 2018
Chronic prenatal nicotine exposure alters enkephalin mRNA regulation in the perinatal rat adrenal medulla	Wong 2003
In utero nicotine exposure promotes M2 activation in neonatal mouse alveolar macrophages	Wongtrakool 2012
Prenatal nicotine exposure alters lung function and airway geometry through alpha7 nicotinic receptors	Wongtrakool 2012
Rat embryogenesis following exposure to alcohol and nicotine	Woo 1988
DNA hypermethylation of acetoacetyl-CoA synthetase contributes to inhibited cholesterol supply and steroidogenesis in fetal rat adrenals under prenatal nicotine exposure	Wu 2016
Gestational nicotine exposure exaggerates hyperthermic enhancement of laryngeal chemoreflex in rat pups	Xia 2010
Prenatal gender-related nicotine exposure increases blood pressure response to angiotensin II in adult offspring	Xiao 2008
Antenatal nicotine induces heightened oxidative stress and vascular dysfunction in rat offspring	Xiao 2011
Perinatal nicotine exposure increases angiotensin II receptor-mediated vascular contractility in adult offspring	Xiao 2014
Antenatal Nicotine Exposure Increases Hypertensive Response in Adult Offspring: Role of ROS	Xiao 2015
Nicotine exposure during pregnancy programs osteopenia in male offspring rats via alpha4beta2-nAChR-p300-ACE pathway	Xiao 2019
Prenatal nicotine exposure intergenerationally programs imperfect articular cartilage via histone deacetylation through maternal lineage	Xie 2018
Fetal and adolescent nicotine administration: effects on CNS serotonergic systems	Xu 2001
Corrigendum to "Nicotine-induced over-exposure to maternal glucocorticoid and activated glucocorticoid metabolism causes hypothalamic-pituitary-adrenal axis-associated neuroendocrine metabolic alterations in fetal rats"	Xu 2013
Prenatal nicotine exposure enhances the susceptibility to metabolic syndrome in adult offspring rats fed high-fat diet via alteration of HPA axis-associated neuroendocrine metabolic programming	Xu 2013
Prenatal nicotine exposure-induced intrauterine programming alteration increases the susceptibility of high-fat diet-induced non-alcoholic simple fatty liver in female adult offspring rats	Xu 2015
Prenatal nicotinic exposure suppresses fetal adrenal steroidogenesis via steroidogenic factor 1 (SF-1) deacetylation	Yan 2014

Augmented autophagy suppresses thymocytes development via Bcl10/p-p65 pathway in prenatal nicotine exposed fetal mice	Yan 2021
Alterations in hippocampal cholinergic receptors and hippocampal behaviors after early exposure to nicotine	Yanai 1992
Nicotine, an alpha7 nAChR agonist, reduces lipopolysaccharide-induced inflammatory responses and protects fetuses in pregnant rats	Yang 2014
Nicotine treatment prolongs gestation and inhibits cervical ripening in pregnant rats	Yang 2014
The protective effect of melatonin in lungs of newborn rats exposed to maternal nicotine	Yildiz 2018
Antioxidant role of melatonin against nicotine's teratogenic effects on embryonic bone development	Yilmaz 2018
Prenatal nicotine exposure induces offspring high response to central angiotensin II. [Chinese]	Yu 2013
Prenatal nicotine exposure results in the myocardial fibrosis in the adult male offspring rats	Yu 2016
Prenatal Nicotine Exposure Results in the Inhibition of Baroreflex Sensitivity Induced by Intravenous Injection Angiotensin II in the Adult Male OffspringRats	Yu 2017
Deficits in development of central cholinergic pathways caused by fetal nicotine exposure: differential effects on choline acetyltransferase activity and [3H]hemicholinium-3 binding	Zahalka 1992
Fetal nicotine exposure alters ontogeny of M1-receptors and their link to G-proteins	Zahalka 1993
Vasopressin system is impaired in rat offspring prenatally exposed to chronic nicotine	Zbuzek 1992
Prenatal nicotine exposure increased duration of nicotine-induced analgesia in adult rats	Zbuzek 1994
Effect of pre- and postnatal nicotine exposure on vasopressinergic system in rats	Zbuzek 1999
Placental mechanism of prenatal nicotine exposure-reduced blood cholesterol levels in female fetal rats	Zhang 2018
MicroRNA-181a down-regulates BKcachannel leading to enhanced coronary vascular tone in perinatal nicotine exposed offspring	Zhang 2018
Prenatal nicotine exposure induces depression-like behavior in adolescent female rats via modulating neurosteroid in the hippocampus	Zhang 2019
Reduced testicular steroidogenesis in rat offspring by prenatal nicotine exposure: Epigenetic programming and heritability via nAChR/HDAC4	Zhang 2020
Repetitive Mild Traumatic Brain Injury in an Awake, Unanesthetized Mouse Model of Perinatal Nicotine Exposure Produces Transient Novelty-Seeking and Depression-Like Behaviors	Zhang 2022
Prenatal nicotinic exposure attenuates respiratory chemoreflexes associated with downregulation of tyrosine hydroxylase and neurokinin 1 receptor in rat pup carotid body	Zhao 2016
From the Cover: Prenatal Nicotinic Exposure Attenuates Respiratory Chemoreflexes Associated With Downregulation of Tyrosine Hydroxylase and Neurokinin 1 Receptor in Rat Pup Carotid Body	Zhao 2016
Bronchopulmonary C-fibers' IL1RI contributes to the prolonged apneic response to intra-atrial injection of capsaicin by prenatal nicotinic exposure in rat pups	Zhao 2016
Prenatal nicotinic exposure upregulates pulmonary C-fiber NK1R expression to prolong pulmonary C-fiber-mediated apneic response	Zhao 2016
Attenuated Tregs increase susceptibility to type 1 diabetes in prenatal nicotine exposed female offspring mice	Zhao 2019

Prolongation of bronchopulmonary C-fiber-mediated apnea by prenatal nicotinic exposure in rat pups: role of 5-HT<inf>3</inf> receptors	Zhao 2019
Prenatal nicotine exposure induces gender-related left ventricular arterial uncoupling in adult offspring	Zhenhua 2014
nAChRs-ERK1/2-Egr-1 signaling participates in the developmental toxicity of nicotine by epigenetically down-regulating placental 11beta-HSD2	Zhou 2018
Two intrauterine programming mechanisms of adult hypercholesterolemia induced by prenatal nicotine exposure in male offspring rats	Zhou 2019
Nicotine administration decreases the number of binding sites and mRNA of M1 and M2 muscarinic receptors in specific brain regions of rat neonates	Zhu 1998
Effects of perinatal nicotine exposure on development of [3H]hemicholinium-3 binding sites in rat neonate brain	Zhu 2000
Prenatal nicotine exposure mouse model showing hyperactivity, reduced cingulate cortex volume, reduced dopamine turnover, and responsiveness to oral methylphenidate treatment	Zhu 2012
A prenatal nicotine exposure mouse model of methylphenidate responsive ADHD-associated cognitive phenotypes	Zhu 2017
Synergistic effects of prenatal nicotine exposure and post-weaning high-fat diet on hypercholesterolaemia in rat offspring of different sexes	Zhu 2019
Maternal nicotinic exposure produces a depressed hypoxic ventilatory response and subsequent death in postnatal rats	Zhuang 2014
Prenatal nicotinic exposure augments cardiorespiratory responses to activation of bronchopulmonary C-fibers	Zhuang 2015
The influence of prenatal nicotine exposure on vascular endothelial function in adult rats offspring	Ziyang 2014
