# Supplementary material for: Developing a taxonomy to describe offspring outcomes in studies involving pregnant mammals’ exposure to non-tobacco nicotine: A systematic scoping review
Source: PLoS One. 2023 Feb 3;18(2):e0280805. doi: 10.1371/journal.pone.0280805 (PMC9897539; doi:10.1371/journal.pone.0280805)
Supplement: Attachment — Submitted filename: Response to reviewers.docx [file pone.0280805.s004.docx]

Dear Editors and Reviewers,

Thank you for you feedback on our manuscript, please find responses and actions taken in relation to your feedback below:

1. Please ensure that your PRISMA flowchart details the reasons for record exclusion at each stage

*The PRISMA diagram now includes human or non-mammalian participants, conference abstracts or reviews, and exposure to nicotine via lactation as reasons for exclusion*

2. Systematic and scoping reviews should provide an up-to-date overview of the research topic covered. Since the literature search was completed over a year ago, we also request that your update your search to be more current.

*The searches were completed again on 21^st^ September 2022 and any new relevant citations have been added to the final taxonomy figures*

3. Please ensure that your manuscript meets PLOS ONE's style requirements, including those for file naming.

*The manuscript has been updated to reflect the guidance provided in your email. File names have also been amended to follow these requirement*

4. In your Data Availability statement, you have not specified where the minimal data set underlying the results described in your manuscript can be found.

*We have now added a supporting information file that includes the title, author and publication year for all citations included in the systematic scoping review*

5. Please review your reference list to ensure that it is complete and correct.

   *All references included in the manuscript have been checked.*

Thank you for taking the time to review our manuscript and please get in touch if you require any further information or changes.

Best wishes,

Lucy Phillips
